# Delamination Detection in CFRP Stringers with A_0_-Mode Lamb Wave Curvature

**DOI:** 10.3390/s25061915

**Published:** 2025-03-19

**Authors:** Gaozheng Zhao, Yike Zeng, Zhenyang Yu, Kaihua Sun, Jian Chen

**Affiliations:** 1 State Key Laboratory of Fluid Power and Mechatronic Systems, School of Mechanical Engineering, Zhejiang University, Hangzhou 310027, China; zhaogaozheng@zju.edu.cn (G.Z.); 12025064@zju.edu.cn (Y.Z.); 12125043@zju.edu.cn (Z.Y.); 2 School of Physics and Electronic Engineering, Fuyang Normal University, Fuyang 236037, China; 3 Institute of Machinery Manufacturing Technology, China Academy of Engineering Physics, Mianyang 621900, China; sunkaihua@caep.cn

**Keywords:** lamb wave, A_0_-mode, CFRP stringer, damage detection, guided wavefield

## Abstract

Ultrasonic detection of delamination in stringer-stiffened panels made of carbon fiber-reinforced plastics (CFRPs) presents a critical challenge due to their complex geometry, complicated properties and large size. In this work, a delamination detection method using the wavefield curvature of the A_0_-mode Lamb wave is proposed. Firstly, the underlying mechanism is numerically investigated to examine the interactions between the A_0_-mode Lamb wave and delamination at different sites of the stringer. Then, the wavefield curvature sensitive to local anomalies is revealed owing to the higher-order derivative nature. Thereafter, the proposed method is utilized to detect delamination in the fabricated CFRP specimens and the results are compared with X-ray computed tomography, confirming the feasibility and effectiveness of the proposed method. This viable method, capable of detecting delamination in larger CFRP stringers, will find great potential in the efficient non-destructive testing of CFRP structures in different applications.

## 1. Introduction

Carbon fiber-reinforced plastics (CFRPs) are widely used in aerospace, the automotive industry, rail transport, infrastructure and renewable energy owing to their distinct advantages, including designable performance, multifunctional compatibility, high specific strength and stiffness, excellent fatigue performance, corrosion resistance and others [1,2]. However, CFRPs generally operate under severe environments, such as high temperatures, humidity, corrosion and hygrothermal aging, which can introduce fiber–matrix damage and therefore affect the overall performance. As a result, the detection of damage in CFRPs is crucial to ensuring structural stability and safety.

As a member of the ultrasonic family, guided waves find wide use in damage detection in CFRPs given their large propagation range, low attenuation, rich modal information and sensitivity to minor damage [3,4,5,6]. Su et al. proposed a guided wave tomography method to detect CFRP damage using the power spectral density of Lamb waves [7]. On the other hand, Sun et al. used a sensor network and proposed an annular probability distribution function based on damage scattering characteristics, which enhanced the off-axis localization and tolerance capabilities of guided Lamb wave imaging [8]. Memmolo et al. detected and assessed the disbonding of CFRP stringers through further exploitation of the arrival time of scattered guided waves [9]. Meanwhile, the investigation and optimization of the configuration and arrangement of piezoelectric (PZT) sensors for Lamb wave excitation have also been conducted [10].

The lowest antisymmetric Lamb wave mode, or A_0_-mode Lamb wave, shows sensitivity to CFRP panel damage [11]. Wilcox et al. demonstrated the larger strain energy density of the A_0_-mode of Lamb wave compared to the S_0_-mode, which resulted in higher sensitivity to delamination damage [12]. Huo et al. obtained a similar conclusion on A_0_-mod Lamb waves during delamination detection in CFRP composites via finite element simulations [13]. Scholars have also focused on noncontact measurement of A_0_-mode Lamb waves with optical detection, which can rapidly obtain the full wave field. Hua et al. proposed a phase unwrapping imaging method for efficient localization and quantification of CFRP damage, featuring dense spatial measurements, which cannot be achieved using conventional piezoelectric transducers, via laser scanning, [14]. Zhang et al. investigated the interaction between A_0_-mode Lamb waves and delamination damage for the optimal extraction of sensitive modes, which were then used for frequency–wavenumber-guided wave imaging [15]. Afterwards, Juarez et al. proposed a multifrequency local wavenumber method to determine CFRP damage size and depth through the correlation of local wavenumbers with theoretical dispersion curves [16]. Meanwhile, Purcell et al. used a three-dimensional (3D) scanning laser Doppler vibrometer to obtain guided wave fields and damage mapping from guided wave mode spectroscopy [17].

However, the abovementioned methods only apply to flat structures and cannot be directly applied to curved structures such as stiffeners [18]. Moreover, although wavenumber methods are advantageous in terms of information richness, their spatial resolution is highly limited. To this end, wavefield curvature, which exhibits high sensitivity to damage in curved CFRPs, provides an alternative solution [19]. On the other hand, owing to the low attenuation of guided waves in propagation, their wavefields can carry abundant structural health information, which can be greatly exploited for damage detection [20,21,22]. To attain such a goal, Saito et al. leveraged the two-dimensional time-slowness maps of A_0_-mode Lamb waves for delamination imaging in L-shaped CFRP structures [23]. Similarly, Sha et al. proposed a wavefield curvature imaging algorithm to explore the anomalous information resulting from the interaction between A_0_-mode Lamb waves and delamination damage [24,25]. Despite their great successes, the traditional methods for full-space damage detection in curved CFRPs using sensor networks remain challenging [26]. Nevertheless, this problem may be addressed by taking advantage of guided wavefields along with optical measurements, i.e., scanning laser doppler vibrometry (SLDV), which requires several PZT actuators to excite guided waves in full space.

In this study, an ultrasonic imaging method for the detection of delamination in CFRP stringers, based on A_0_-mode Lamb waves, was proposed. A single PZT actuator was used to generate guided wavefields across all regions of the CFRP stringers, which were then densely acquired with SLDV. A window function was introduced to capture the A_0_-mode Lamb waves while eliminating boundary reflections, which render the highlighted damaged areas. Finite element simulations and experiments were conducted to demonstrate the feasibility of the proposed method and the results were comparatively verified via X-ray computed tomography (CT). The simulated and experimental results showed good consistency, which confirms the effectiveness of the proposed method for delamination detection in CFRP stringers. Overall, this study presented efficient and large-scale inspection of damage in CFRP stringers, which may open an important avenue towards quality control of CFRP components in various fields.

## 2. Imaging Method

Ultrasonic wave propagation can be considered to be energy transmission from actuating sources, and the presence of CFRP damage alters energy distribution. Here, the ultrasonic signals corresponding to the wavefield were recorded in 3D format; that is, $v\left[x,y,t\right]$, with *x* and *y* denoting the numbers of measuring points in the *x* and *y* directions in space and *t* being time. For this objective, the wavefield of A_0_-mode Lamb waves at various time stamps can be represented as follows:

(1) $$v_{A_{0}}\left[x,y,T_{A_{0}}\right]=v\left[x,y,t\right]W\left(T_{A_{0}}\right)$$

where $W\left(T_{A_{0}}\right)$ indicates the window function:

(2) $$W\left(T_{A_{0}}\right)=\left\{\begin{array}{cc} 0 & t<t_{A_{0}}-T_{A_{0}}/2\\ 1 & t_{A_{0}}-T_{A_{0}}/2\leq t\leq t_{A_{0}}+T_{A_{0}}/2\\ 0 & t_{A_{0}}+T_{A_{0}}/2<t \end{array}\right.$$

with $t_{A_{0}}$ and $T_{A_{0}}$ representing the peak time of the A_0_-mode Lamb wave and the window width, respectively.

Then, curvatures are used mainly because of their higher-order derivative nature, which is sensitive to local anomalies in wavefield signals associated with damage. The wavefield curvature $w_{A_{0}}\left[x,y,T_{A_{0}}\right]$ can be derived using the central difference in the wavefield signal as follows:

(3) $$\begin{array}{c} w_{A_{0}}\left[x,y,T_{A_{0}}\right]=\left[\left(v_{A_{0}}\left[x+1,y,T_{A_{0}}\right]-v_{A_{0}}[x,y,T_{A_{0}}]\right)-\left(v_{A_{0}}\left[x,y,T_{A_{0}}\right]-v_{A_{0}}[x-1,y,T_{A_{0}}]\right)\right]/\Delta x^{2}\\ +\left[\left(v_{A_{0}}\left[x,y+1,T_{A_{0}}\right]-v_{A0}[x,y,T_{A_{0}}]\right)-\left(v_{A_{0}}\left[x,y,T_{A_{0}}\right]-v_{A_{0}}[x,y-1,T_{A_{0}}]\right)\right]/\Delta y^{2} \end{array}$$

where Δx and Δy denote the grid spacings along the *x* and *y* axes, respectively.

Finally, the images of wavefield curvature within the time window were amalgamated, and a comprehensive energy map was rendered as follows [24]:

(4) $$EMAP_{A_{0}}\left[x,y\right]=\sum_{t_{A_{0}}-T_{A_{0}}/2}^{t_{A_{0}}+T_{A_{0}}/2}w_{A_{0}}^{2}\left[x,y,T_{A_{0}}\right]$$

where $EMAP_{A_{0}}\left[x,y\right]$ denotes the spatial distribution of energy.

**Figure 1:**
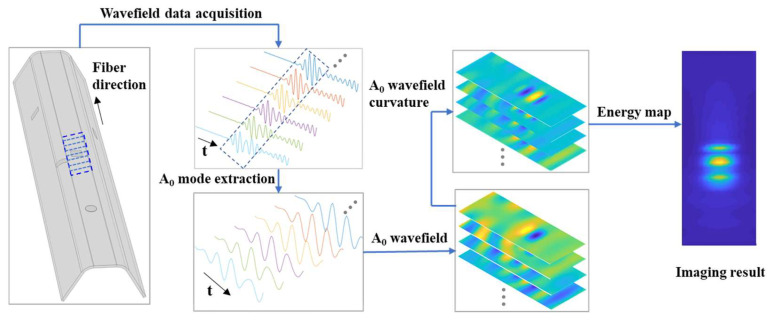
The schematic flowchart of damage detection with wavefield curvature.

For clarity, the flowchart of damage detection with wavefield curvature of the A_0_-mode Lamb wave is briefly summarized and outlined in Figure 1.

## 3. Simulations

### 3.1. Simulation Model

**Figure 2:**
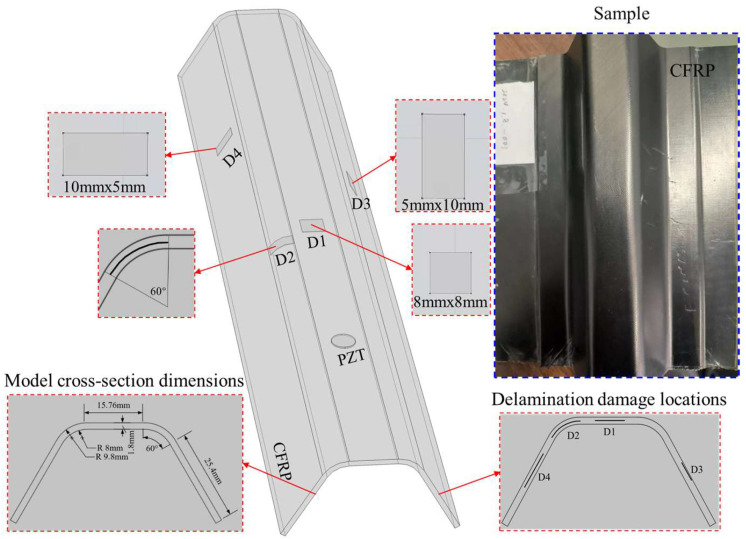
The schematic geometry of the CFRP stringer and damage locations in the simulation model.

**Figure 3:**
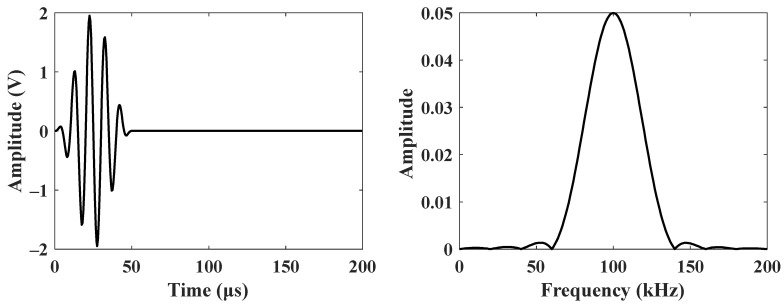
The signal for guided wave excitation.

Firstly, finite element simulations were conducted to validate the feasibility of A_0_-mode Lamb waves for the inspection of full-space damage in CFRP stringers. COMSOL Multiphysics 5.6 was utilized to establish a 3D finite element simulation model consisting of Solid Mechanics, Electrostatics, and Electrical Circuit modules. Among them, the Solid Mechanics module accounts for the mechanical behavior of carbon fiber-reinforced plastic (CFRP) structures and lead zirconate titanate (PZT) structures. Notably, piezoelectric material is assigned to PZT structures to simulate the piezoelectric effect. Meanwhile, the Electrostatics module tackles the electrical characteristics of PZT structures, while the Electrical Circuit module provides the excitation voltage input. This configuration enables generating ultrasonic Lamb waves directly from PZT excitation and seamlessly coupling to the CFRP structure. Figure 2 illustrates the geometry of the CFRP stringer and the preset damage. Notably, the length and thickness of the CFRP stringer were 300 mm and 1.8 mm, respectively, and the ply direction was uniformly set as 0°. All the areas of damage, which were marked as D1, D2, D3 and D4, had the same thickness of 0.2 mm. Table 1 provides detailed parameters on material properties. During the guided wave excitation, the PZT actuator was bonded to the top surface of the stringer, which was then fed with an electrical signal from a five-cycle sine wave modulated by a Hanning window (Figure 3). For experimental measurements in the following context, the central frequency of the guided wave was set to 100 kHz. To avoid distortion on guided wave propagation, the largest mesh size was set to be smaller than λ/6 (λ is the wavelength of an A_0_-mode Lamb wave). Herein, the calculated λ was 20 mm, given the excitation frequency of 100 kHz; accordingly, the mesh sizes for the stringer and PZT actuator were set as 2 and 1 mm, respectively [27]. Meanwhile, wave propagation in the whole stringer was accommodated by setting the simulation time to 200 µs with a step size of 1 µs.

### 3.2. Simulation Results

**Figure 4:**
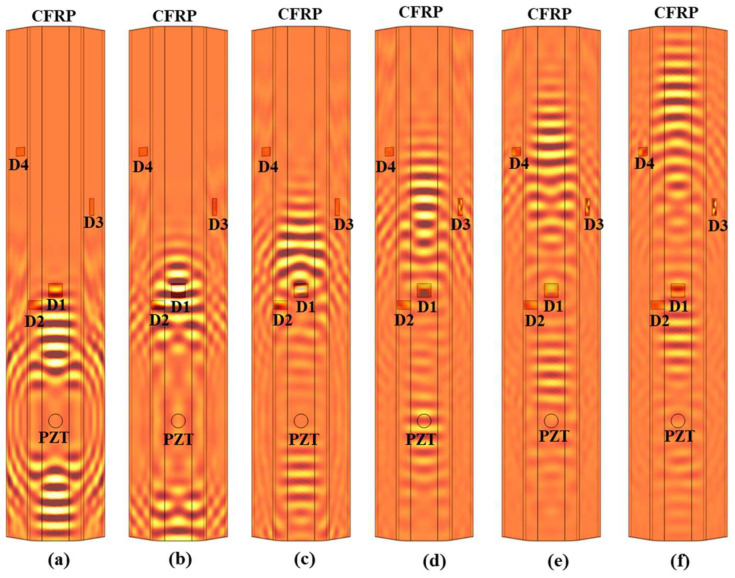
Propagation of simulated Lamb wave in the CFRP stringer: (**a**) time = 60 µs, (**b**) time = 80 µs, (**c**) time = 100 µs, (**d**) time = 120 µs, (**e**) time = 140 µs, and (**f**) time = 160 µs.

To clarify the interactions between Lamb waves and damage locations, Lamb wave propagation in the CFRP stringer is illustrated (Figure 4). Specifically, the A_0_-mode was dominated by out-of-plane displacements and the S_0_ mode by in-plane displacements [28]. Given that the SLDV, which is used for dense-wavefield measurement, is only sensitive to out-of-plane displacements, the A_0_-mode Lamb wave was considered herein [29]. The A_0_-mode Lamb wave exhibited noticeable interactions with damage locations D1 and D2 in the top regime and standing wave patterns formed after the Lamb wave passed through (Figure 4e). Meanwhile, as the Lamb wave travelled along the stringer side, standing wave patterns were also formed (Figure 4f). However, the standing wave at around D3 was more pronounced than that at D4, mainly because D3 was shallower and the wave energy can be more effectively concentrated within the narrow cavity.

**Figure 5:**
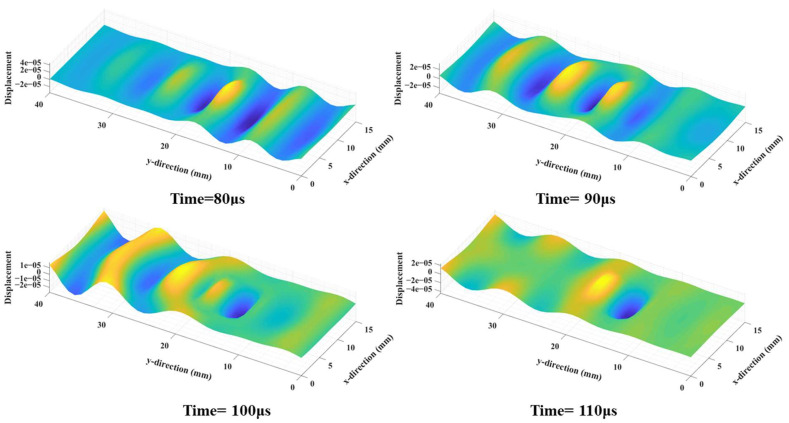
The wavefield of the A_0_-mode Lamb wave around damage location D1 at different times.

**Figure 6:**
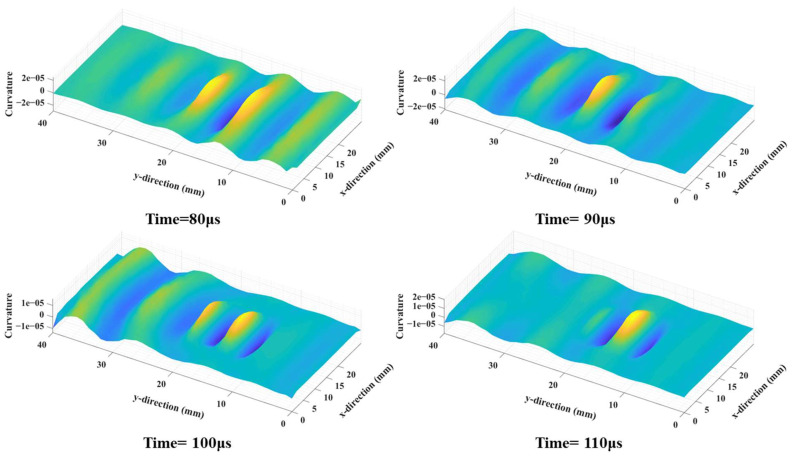
The wavefield curvature maps around damage location D1 at different times.

Next, the wavefields around the damage locations were extracted for further processing. Figure 5 depicts the wavefield evolution of the A_0_-mode Lamb wave during its passage through damage location D1. Notably, the guided wave resonated with the damage at the structural discontinuity, which in turn led to changes in the frequency of standing waves [30]. Then, the curvature maps were derived using the wavefield curvature function, which greatly suppressed the wavefields in normal areas and therefore highlighted the damaged ones (Figure 6).

**Figure 7:**
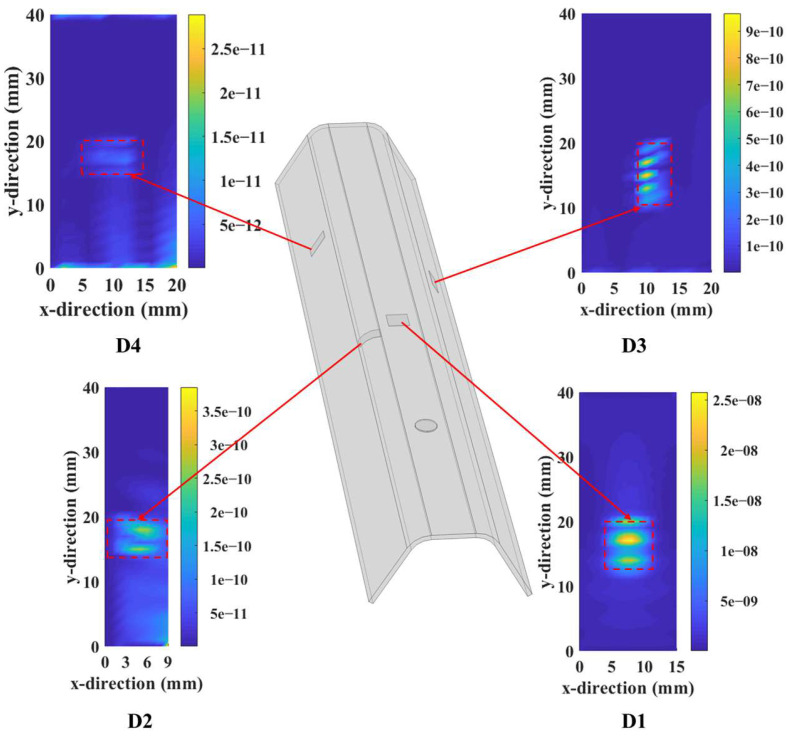
Maps of standing wave energy around the damage locations.

Thereafter, the wavefield curvature maps were added together to construct an energy map of standing waves, and the results obtained for the four damage locations are demonstrated in Figure 7. For clarity, the energy maps were normalized with respect to their maximum value. From the figure, the proposed method can visualize well the damage locations with distinct contours, and the findings are close to those of artificial settings in simulation. This can be anticipated given that wavefield curvatures are sensitive to local anomalies in wavefield signals due to their high-order derivative nature.

**Figure 8:**
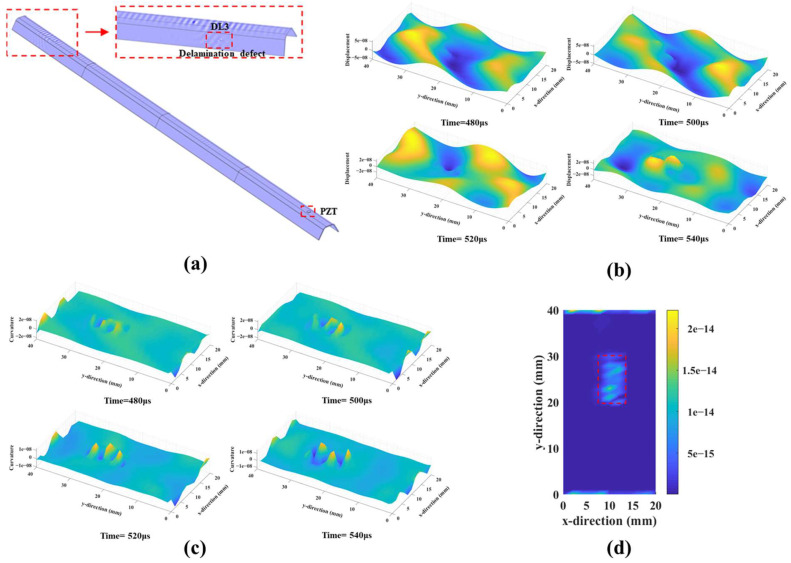
(**a**) The schematic simulation model for a prolonged stiffener; (**b**) the wavefield of the A_0_-mode Lamb wave at different times; (**c**) the wavefield curvature of the A_0_-mode Lamb wave at different times; and (**d**) the simulated damage imaging result.

For convenience, we performed a numerical simulation on a prolonged stiffener with a length of 1.2 m, in which a damage location was 1 m away from the PZT actuator (Figure 8a). For clarity, the evolution of the interaction between the A_0_-mode Lamb wave and the damage location was visualized (Figure 8b,c). A pronounced standing wave evidently formed around the damage location, and the proposed method can clearly illustrate the damage despite its distant location (Figure 8d).

## 4. Experiments

### 4.1. Experimental Setup

**Figure 9:**
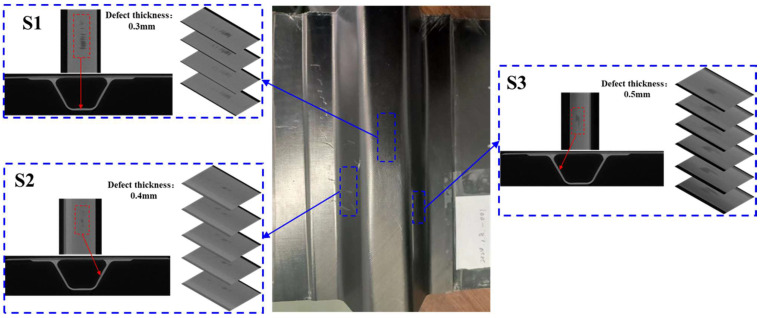
CT damage images of CFRP stringer.

**Figure 10:**
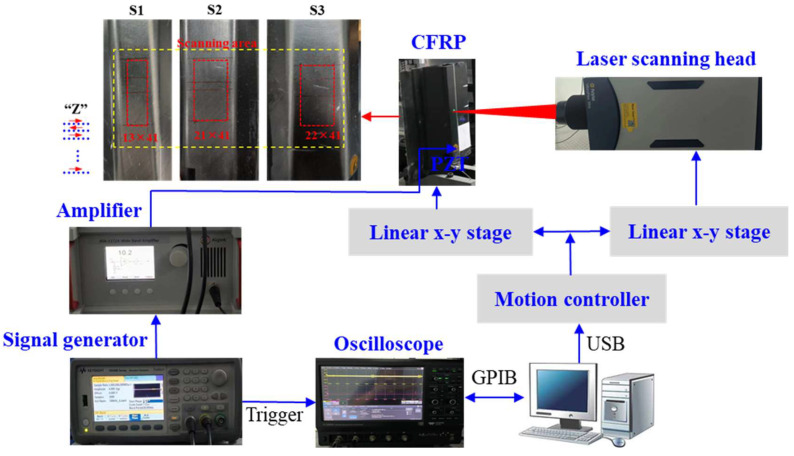
Experimental setup.

Experiments were conducted on the fabricated CFRP stringers to further verify the effectiveness of the proposed method (Figure 9). For consistency with the simulations, the dimensions of the fabricated CFRP stringers were the same as in simulations. Notably, to facilitate practical applications, damage locations were intentionally introduced by varying the parameters of the manufacturing process, rather than artificial defects being embedded. The fabricated CFRP stringers involved designing the structure, selecting the materials, preparing the mold, and laminating the carbon fiber fabric by hand layup, followed by resin injection via a hot-press tank process. The resin is then cured under specific heat and pressure to form a strong composite, after which the panels are demolded and trimmed. Delamination damage can occur during this process. Three damage locations (S1, S2 and S3) existed in the fabricated specimen, as evidenced by X-ray CT (Figure 9). Then, an experimental setup was built as shown in Figure 10. A PZT actuator was attached to the top surface of the specimen for Lamb wave propagation across the stringer. As in the simulation, the PZT actuator was excited with a five-cycle sine wave modulated by a Hanning window. A 100 kHz centre frequency and 40 V applied voltage were used, respectively. The Lamb waves were then remotely detected using the SLDV (Polytech GmbH, Baden-Württemberg, Germany), which comprised an optical sensor head and a digital controller. To maximize the detection sensitivity, the optical sensor head was accurately positioned to ensure the laser beam was aligned perpendicular to the surface of the examined specimen. Meanwhile, the SLDV was mounted on a 2D stage to enable detection beam scanning. On the other hand, the output signal from the SLDV controller was sent to an oscilloscope (Lecroy HDO6054, Teledyne Technologies, Thousand Oaks, CA, USA) with a sampling rate of 100 MHz, in which the waveforms were averaged over 50 repetitions to improve the signal-to-noise ratio. For simplicity, the wavefields were acquired around the damage locations. Herein, for simplicity, the wavefields were sampled around the damage locations by raster scanning with the use of the SLDV, with a step size of 1 mm along the *x* and *y* directions. The dimensions of the scanning area are 12 mm × 40 mm (S1), 20 mm × 40 mm (S2) and 21 mm × 40 mm (S3), within which the data are raster collected with a step size of 1 mm, and the total collection points are 533 (S1), 861 (S2) and 902 (S3).

### 4.2. Damage Imaging Results

**Figure 11:**
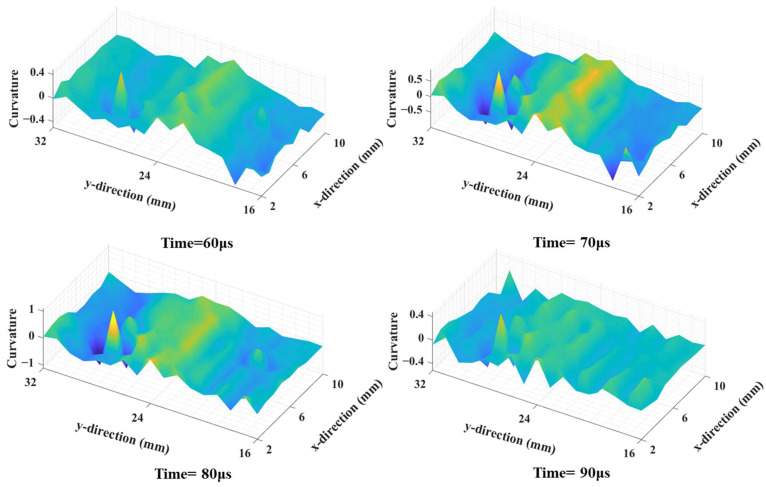
The representative evolution of A_0_-mode wavefield curvature for damage location S1 at different times.

**Figure 12:**
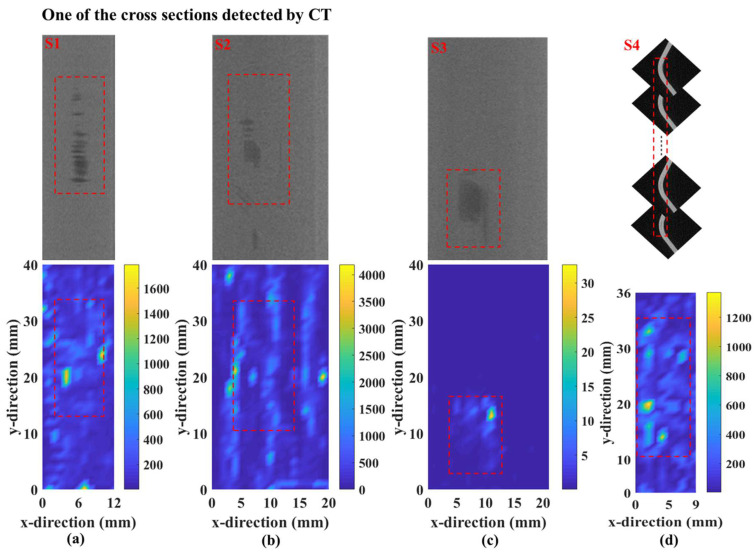
Damage imaging results of (**a**) S1, (**b**) S2, (**c**) S3 and (**d**) S4.

In the acquired time–space guided wavefield, the proposed method was used to extract curvature information on various damage locations. Figure 11 shows the evolution of the A_0_-mode Lamb wave interacting with damage location S1. As observed in the simulations, standing waves were formed at the damage location edges, and the imaging results with the wavefield curvature effectively highlighted the damaged areas, as shown in Figure 12a. However, the measured damage reasonably deviated from the CT image, particularly in the central part. Such discrepancies mainly arose from the damage irregularity in morphology and distribution; i.e., the standing wave energy on the middle part of damage location S1 was considerably weaker given that the damage depth in the detection surface at this location was considerably larger than that at the edges [10]. Moreover, given the presence of irregularities, the interaction between the Lamb waves and damage cannot yield strong standing wave energy.

Similarly, standing waves were evidently formed when the A_0_-mode Lamb wave passed through damage locations S2 and S3 on the side surfaces of the stringer. Accordingly, the wavefield curvature changed considerably in the damaged regions, and the energy map distributions highlight the damage at locations S2 and S3 (Figure 12b and Figure 12c, respectively). Notably, compared with damage location S2, the damage at S3 was more prominent given that it had a more intact morphology and was located closer to the detection surface, as indicated by the CT images.

On the other hand, the proposed method can also be applied to damage detection on stringer corners [31]. The same as above, when the Lamb wave passed through the damage on the curved surfaces, an evident standing wave resulted from their interaction. Thereafter, the wavefield curvature was calculated to derive the energy map distribution, and the damage was magnified (Figure 12d).

To date, the wavefield curvature of A_0_-mode Lamb waves can effectively realize imaging of damage in the full space of stringers, either on the top and side surfaces or the corners. In addition, the damage locations in the fabricated CFRP stringer were produced by varying the manufacturing process, which did not possess the complete and clear contours as those in the simulation. As a result, compared with the simulated findings, the results of experimental imaging were not very significant.

## 5. Discussion and Conclusions

In addition to the full-space imaging capabilities of CFRP stringers, the working range of the proposed method was further investigated. The distinct advantage of guided waves lies in their long-distance propagation with minimal attenuation. Most reinforced CFRP structures, such as aircraft fuselage panels, exhibit large-aspect ratios of stiffeners, and the guided waves can leverage these strengths. In addition, the proposed method can benefit from great adaptability to complex structures and rapid scanning during optical detection.

Despite the success of full-space damage imaging of CFRP stringers, the proposed method showed poor performance with deeply buried damage. This finding was primarily due to the varying strengths of standing wave energy formed by the interaction of Lamb waves and damage locations at various depths [32]. Particularly, when the damage was far below the detection surface, weak standing-wave energy was exhibited on the detection surface [10,33]. In addition, noise and surface roughness further deteriorated the detected signals, which made the extraction of effective information challenging. This is because the roughness of the structural surface affects the signal-to-noise ratio of the SLDV detection signal and increases the feature extraction of damage-related response information [34].

This study proposes a delamination detection method for stiffened CFRP stiffeners using the wavefield curvature of A_0_-mode Lamb waves. Finite element simulations were conducted to investigate the interaction between the A_0_-mode Lamb wave and delamination damage. Further, simulations and experiments, both of which can effectively visualize the delamination damage as desired, were carried out to verify the effectiveness of the proposed method. Finally, the feasibility of guided wavefields for long-distance damage detection was analysed. These findings demonstrate full-space damage imaging, along with the guided wavefield information, which could offer insights into delamination detection of CFRP stiffeners with high efficiency and convenience.

For random, indeterminate and discontinuous delamination damage, the interaction between guided waves and such defects typically results in weak standing-wave energy, rendering the extraction of characteristic parameters challenging. Consequently, the localization of small-scale, discontinuous delamination damage becomes unfeasible using conventional approaches. To address this limitation, one viable strategy is to enhance the amplitude of guided wave excitation. By doing so, the intensified guided wave can interact more effectively with the random, indeterminate, and discontinuous delamination damage, inducing the generation of higher-order harmonics. These nonlinear features can then be leveraged for delamination damage imaging by extracting and analyzing the corresponding frequency domain characteristics, thereby enabling improved detection and localization of such complex defects.

## Figures and Tables

**Table 1:** Parameters of CFRP properties.

E11/GPa	E22/GPa	E33/GPa	G12/GPa	G13/GPa	G23/GPa	v12	v13	v14
148.67	9.29	9.29	8.48	8.48	5.7	0.27	0.27	0.41

## Data Availability

All data are contained within the article.
